# Phycochemical Constituents and Biological Activities of *Fucus* spp.

**DOI:** 10.3390/md16080249

**Published:** 2018-07-27

**Authors:** Marcelo D. Catarino, Artur M. S. Silva, Susana M. Cardoso

**Affiliations:** Department of Chemistry & Organic Chemistry, Natural Products and Food Stuffs Research Unit (QOPNA), University of Aveiro, Aveiro 3810-193, Portugal; mcatarino@ua.pt (M.D.C.); artur.silva@ua.pt (A.M.S.S.)

**Keywords:** *Fucus* spp., nutritional value, bioactivities, fucoidan, phlorotannins, fucoxanthin

## Abstract

Seaweeds are known to be a good supply of key nutrients including carbohydrates, protein, minerals, polyunsaturated lipids, as well as several other health-promoting compounds capable of acting on a wide spectrum of disorders and/or diseases. While these marine macroalgae are deeply rooted in the East Asian culture and dietary habits, their major application in Western countries has been in the phycocolloid industry. This scenario has however been gradually changing, since seaweed consumption is becoming more common worldwide. Among the numerous edible seaweeds, members of the genus *Fucus* have a high nutritional value and are considered good sources of dietary fibers and minerals, especially iodine. Additionally, their wealth of bioactive compounds such as fucoidan, phlorotannins, fucoxanthin and others make them strong candidates for multiple therapeutic applications (e.g., antioxidant, anti-inflammatory, anti-tumor, anti-obesity, anti-coagulant, anti-diabetes and others). This review presents an overview of the nutritional and phytochemical composition of *Fucus* spp., and their claimed biological activities, as well as the beneficial effects associated to their consumption. Furthermore, the use of *Fucus* seaweeds and/or their components as functional ingredients for formulation of novel and enhanced foods is also discussed.

## 1. Introduction

Seaweeds, i.e., marine macroalgae, have a long tradition of usage and applications among the far East populations, either for direct consumption and nutrition or for medicinal purposes, whereas, in Western countries, their industrial utilization has been rather confined to extraction of phycocolloids and, to a lesser extent, certain fine biochemicals [1]. Nevertheless, this panorama is currently shifting as macroalgae are becoming more and more popular since, in addition to not competing with food crops for the use of arable land and fresh water resources, they are claimed to be a good supply of key nutrients including carbohydrates, protein and minerals [2], as well as a rich source of health-promoting compounds capable of acting on a wide spectrum of disorders and/or diseases [3]. Note also that the Japanese have the world’s longest life expectancy, which has been partly associated with their different dietary patterns, and of course, the regular consumption of macroalgae [4]. Based on this evidence, marine macroalgae are presently pointed out as the plant-based food of the future, and their regular consumption among Western population has been trending upward [5]. Indeed, the global functional food market, which was valued at US $168 in 2013 and expected to exceed US $305.4 billion by 2020, is believed to be one of the most important exploiting opportunities where direct applications of seaweeds, crude extracts or purified fractions of seaweeds might hold potential [4].

**Figure 1:**
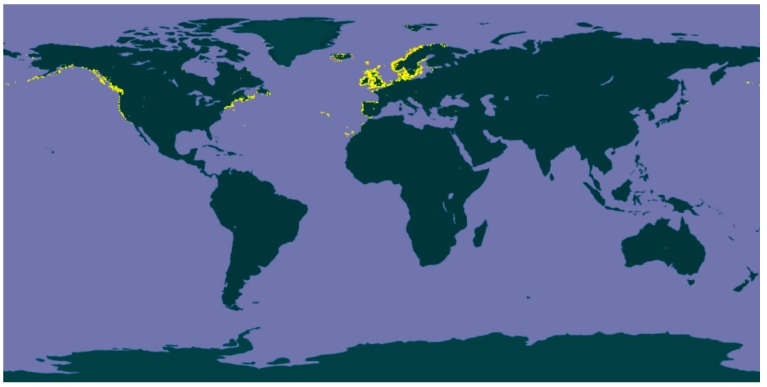
Geographical distribution of *Fucus* L. © OpenStreetMap contributors (licensed under Open Data Commons Open Database License); GBIF.org (25 July 2018) GBIF Occurrence Download https://doi.org/10.15468/dl.l5mbpr (licensed under CC BY 4.0).

*Fucus*, an abundant and widely distributed genus of brown, perennial and edible seaweeds, has earned increasing attention throughout the last years. This genus occupies the cold-temperate waters from the littoral and sublittoral regions along the rocky shorelines of the northern hemisphere (Figure 1) [6], and is comprised of 66 currently accepted taxonomically species all characterized by a greenish brown trisected thallus, i.e., a structure consisting of a holdfast, a small stipe and flattened dichotomously-branched blades with terminal receptacles that swell during the reproductive season. The blades usually have a central-thickened area called midrib and, in some species, air bladders can be found to keep them floating in a vertical position when submerged [7].

*F. vesiculosus* is the most well-known species from *Fucus* genus, often dominating shallow macroalgae communities. It grows on the mid-tide zone of high salinity waters forming large belts that have an important role in the structure of the habitats that harbor species-rich epiphytic and epibenthic communities [8]. Other important species from this genus include *F. spiralis* and *F. serratus*, the former growing in the upper intertidal zone and the latter in the lower-mid intertidal zone [9,10].

With high content in dietary fiber, minerals and vitamins, and low in fat, seaweeds belonging to this genus have an exceptional combination of macro- and micronutrients that make them very interesting from a nutritional perspective. In fact, several *Fucus* spp. have long been harvested and used as food sources mainly in far East Asian countries, but also in some coastal countries of Western Europe and Alaska [11]. In particular, *F. spiralis* is a popular delicacy in the Azores Islands where its swollen receptacles are very appreciated and known as sea lupines [12].

In traditional medicine, *Fucus* spp. became very popular mainly due to their high content in iodine, which renders them remarkable therapeutic properties for treating goiter, i.e., the swelling of thyroid and thyroid-related complications, and obesity [13]. Moreover, this genus is also an excellent source of bioactive compounds such as fucoidans, phlorotannins and fucoxanthin, which have been repeatedly shown to possess important therapeutic properties including the treatment of cellulite, blood clot formations, rheumatoid arthritis, asthma, atherosclerosis, diabetes, psoriasis and skin diseases, cancer and other oxidative and inflammatory related conditions [14,15,16,17]. Thanks to the presence of such functional compounds, *Fucus* spp. have been seen as potential functional and/or active ingredients with great interest and applicability not only in the cosmetic and pharmaceutical industries but also in the food and nutraceutical industries [18]. Indeed, the development of novel and improved foods containing seaweeds and/or seaweed-derived components is already a reality that is showing promising results, mainly in the field of dayries and seafoods [19,20,21,22].

In this context, the present manuscript reviews relevant studies on the nutritional and phytochemical composition of *Fucus* spp., highlighting the health benefits of its consumption and the main claimed benefic effects of their major bioactive metabolites. Additionally, the use of whole *Fucus* seaweeds and/or their components as functional ingredients for the development of novel and enhanced foods is discussed.

## 2. Nutrient Composition of *Fucus* Spp.

The moisture content of fresh *Fucus* spp. is very high, reaching up to 88% of the biomass (Table 1). The values are however lower than several other species such as *Laminaria japonica* or *Porphyra yezoensis*, which can reach 94% moisture content [23]. Similar to other seaweeds, *Fucus* spp. contain several nutritional elements important for human body’s physiological functions [24]. However, the concentrations of such elements are very susceptible to seasonality, environmental conditions, geographical origin and several other factors making generalizations of algal composition very difficult. 

### 2.1. Carbohydrates: Dietary Fiber and Polysaccharides

Therefore, one should highlight that some data presented in Table 1 might provide the concentration as a snapshot and not consider the seasonal variations. Similar to other macroalgae, carbohydrates are the most abundant element of *Fucus* spp., serving different roles including storage, mucilage and structural functions [39]. Within this genus, the contents may vary from 26% DW, which was the lowest value registered in *F. serratus*, to 66% DW, as registered in *F. vesiculosus* (Table 1). This high content in carbohydrates does not correspond to high caloric values though, since most of these consist in dietary fibers, i.e., polysaccharides that are not digestible or absorbed in the human gastrointestinal tract [40]. Therefore, species from *Fucus* are a great source of dietary fiber that may play a relevant role for the improvement of human’s gastrointestinal health by regulating the intestinal flow, stimulating the growth of favorable microbiota and preventing colon cancer [12]. Moreover, in combination with high glycemic foods, these fibers may reduce the glycemic response contributing for the regulation of the blood cholesterol and sugar, and ultimately preventing the development of obesity, diabetes, hypercholesterolemia and other related complications [41]. 

As the main constituents of *Fucus* dietary fiber, one can highlight three distinct polysaccharides, namely fucoidan, alginic acid and laminaran (Table 2), albeit their content is very susceptible to inter-species or even intra-species variations.

#### 2.1.1. Alginic Acid

Regardless that variability, alginic acid is generally the most abundant of these polysaccharides, with values reaching up to 59% DW in *F. vesiculosus* [53]. These polysaccharides are normally present in the structure of the cell walls where they play an important structural role, contributing for algae flexibility. Therefore, the more exposed seaweeds are to waves and turbulent conditions, the greater their alginic acid production [59]. Furthermore, concentrations of alginic acid in *F. vesicuslosus*, *F. serratus* and *F. distichus* were found to be 2–3 times higher in the summer (23.97%, 21.80% and 20.60% DW, respectively) than in the spring (8.40%, 10.45% and 9.55% DW, respectively), indicating that the accumulation of these polysaccharides in this genus is highly dependent on seasonality [42]. 

**Figure 2:**
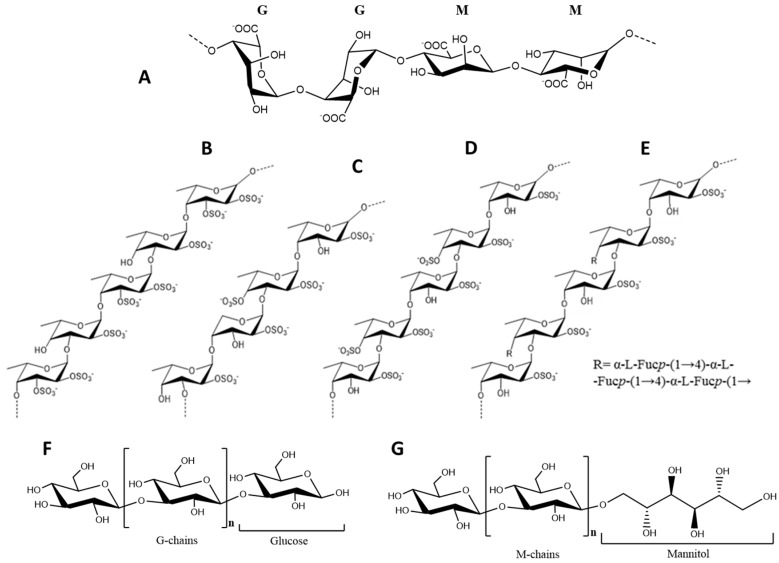
Representative chemical structures of the three main polysaccharides from *Fucus*: (**A**) alginic acid; (**B**–**E**) fucoidans from *F. vesiculosus*, *F. evanescens*, *F. distichus* and *F. serratus*, respectively (without representation of acetate groups); (**F**) G-laminaran; and (**G**) M-laminaran. Structures redrawn from [3].

In terms of structure, alginates are polyuronic saccharides consisting essentially of β-d-mannuronic (M) and α-l-glucuronic (G) acid units linked together by (1→4) bonds, arranged in heteropolymeric (MG) and/or homopolymeric (M or G) (Figure 2A). The mannuronic acid residues establish β-(1→4) linkages that confer a linear and flexible conformation to the M-block segments, whereas guluronic acid forms α-(1→4) linkages that cause G-block segments to have a folded and rigid structural conformation, granting the polymer of a pronounced stiffness [60]. Moreover, in the presence of divalent ions such as Ca^2+^, G-blocks form “egg-box” junctions, allowing the bridging of two antiparallel chains and conferring gel-forming properties to alginates. Therefore, the length of G-blocks in this polysaccharide is determinant for the mechanical and functional properties of the gels [61].

Due to their excellent gel properties, as well as stabilizing and water-holding capacities, alginates have become a very important industrial product in several fields, including textile, material, cosmetic and medical/pharmaceutical, but above all, in food industry where it is mostly used as thickeners, gels, emulsifiers and stabilizers for improving the textural quality of several products such as salad dressings, ice-creams, beers, jellies, lactic drinks and many others [62]. Furthermore, due to their remarkable chelating abilities, biocompatibility, biodegradability and non-antigenicity a growing interest has recently emerged to use these polysaccharides in a multiple biomedical applications [63].

Dietary alginates have been recognized to have a number of potentially beneficial physiological effects in the gastrointestinal tract, contributing for the regulation of the intestinal flow while stimulating the growth of favorable microbiota [41]. Moreover, alginates have the capacity to adsorb a wide range of potential food and chemical mutagens, thereby not only lowering colonic exposure to these agents, but also preventing their absorption into the blood circulation [64,65]. Other common applications for alginates include the symptomatic treatment of heartburn and esophagitis [66], and appetite regulation through modulation of gastrointestinal signals that control hunger, satiety, and food intake [67,68]. Some clinical studies also demonstrated interesting antihyperglycemic and anti-hypercholesterolemic effects on patients administrated with alginic acid formulations, suggesting that consumption of alginates or alginate-rich foods might hold potential for the prevention and/or management of obesity, type 2 diabetes, cardiovascular disease and metabolic syndrome [69,70,71,72,73]. 

Alginates from *Fucus* spp., however, have low G-block ratios (22%) and long M-blocks, giving them poor gel-forming capacity, thus producing low-strength gels [36,74]. These characteristics also greatly decreases the biological and chemical properties of *Fucus* alginates making them less interesting when comparing to others with high G-block content such as those from *Laminaria hyperborean*, *Lessonia trabeculata* or *Saccharina latissima* [75]. In addition, *F. vesiculosus* alginates are particularly sensitive to high temperatures and, according to Truus et al. [76], even a slight rise during the treatment of algal biomass led to a drastic fall in its viscosity. Therefore, algae from this genus are not the typical sources for alginate extraction. 

#### 2.1.2. Fucoidans

In turn, fucoidans are typically extracted from *Fucus* spp. where they also play an important structural role and a protective function against desiccation [3,36]. These polysaccharides are particularly abundant in *F. vesiculosus*, which can accumulate up to 26% DW (Table 2). Chemically, fucoidans are very complex polysaccharides, ranging from 100 to 1600 kDa, being composed mainly of fucose and sulfate, although other monosaccharides (mannose, galactose, glucose, xylose, etc.), uronic acids or even acetyl groups and proteins may be present [77,78]. Regardless, the high variability inherent to these polysaccharides, the structure of fucoidans typically consist of a backbone of (1→3)- and (1→4)-linked α-L-fucopyranose residues, which can be separated in two types: type I consisting of long chains of (1→3)-linked α-L-fucopyranose residues and type II consisting of alternating (1→3)- and (1→4)-linked α-L-fucopyranose residues [15]. Fucoidans from *Fucus* spp. have a type II arrangement and are essentially composed of fucose and sulfate, although small amounts of other monosaccharides may occur.

Fucoidan from *F. vesiculosus* was firstly described as a linear chain of (1→2)- linked 4-*O*-sulfated fucose residues. However, further works have revised this structure and ultimately establishing it as a type II core motif with sulfation occurring at the position C-2 of the 3-linked fucose residues and positions C-2 and -3 of the 4-linked fucose residues (Figure 2B) [79].

Other structures have been reported for *F. evanescens*, *F. distichus* and *F. serratus* fucoidans. According to Bilan et al. [48] the fucoidan from the former has a type II backbone of L-fucose 2-sulfate residues, with additional sulfate and acetyl groups randomly occupying the C-4 positions of the 3-linked residues (Figure 2C). In turn, fucoidan from *F. distichus* consists of repeating units of alternating α-(1→3)- and α-(1→4)-linked l-fucose 2,4-disulfate and only few acetylations in random positions of some residues (Figure 2D) [57]. From this group, only the fucoidan of *F. serratus* has a branched structure (Figure 2E). The backbone of this polysaccharide (also Type II) has half of the 3-linked residues substituted in the position C-4 by α-(1→3)- and α-(1→4)-linked trifucoside units. Sulfate groups can be found mainly in position C-2 and occasionally C-4, although 3,4-diglycosylated and some terminal fucose residues might be non-sulfated. Acetylation may occur in the positions C-3 and C-4 of the 4- and 3-linked fucose residues, respectively. Traces of xylose and galactose may also be present [56]. Naturally, such variability is reflected by different biological potencies among these polysaccharides. 

The ingestion of fucoidans from *Fucus* spp. origin have also been associated with several health benefits. Indeed, studies suggest that *Fucus* spp. fucoidans have potential to counteract oxidative stress and pro-inflammatory events, which are two interdependent pathophysiological processes responsible for the onset of numerous diseases. According to several authors, these polysaccharides may act as antioxidants either by directly scavenging reactive oxygen species such as hydroxyl (OH^●^), peroxyl (ROO^●^) and superoxide anion radicals (O_2_^●−^) [43,80,81], or by stimulating the activity of cellular endogenous antioxidant defenses including superoxide dismutase (SOD), catalase (CAT), glutathione peroxidase (GSH-px), glutathione reductase (GSH-red), glutathione transferase (GSH-tr) and glucose-6-phosphate dehydrogenase [82]. Indeed, in LPS-stimulated BV2 microglial cells treated with *F. vesiculosus* fucoidan, the expression of several pro-inflammatory mediators including nitric oxide (NO^●^), prostaglandin E_2_ (PGE_2_) tumor necrosis factor-α (TNF-α), interleukin-1β (IL-1β), nitric oxide synthase (iNOS), cyclooxygenase-2 (COX) and monocyte chemoattractant protein-1 (MCP-1) were found dose-dependently decreased. Moreover, this polysaccharide exhibited promising inhibitory effects against important pro-inflammatory signaling pathways, through suppression of nuclear factor-κB (NF-κB), protein kinase B (Akt), extracellular signal-regulated kinase (ERK), c-Jun *N*-terminal kinase (JNK), and p38 mitogen-activated protein kinase (p38-MAPK) [83]. 

The anti-inflammatory properties of dietary fucoidans were even demonstrated in some in vivo studies. According to Kuznetsova et al. [84], the serum levels of TNF-α, IL-1 and IL-6 in LPS-injected Balb/c mice were significantly reduced in the animals that have been orally administrated with 50 mg/kg body weight of *F. evanescens* fucoidan. Similarly, the expression of IL-6 was found decreased on colitis-induced Balb/c mice fed with *F. vesiculosus* fucoidan-enriched meals [85], while in a model of gastric ulceration, the oral administration of 20 mg/kg body weight of this polysaccharide to Wistar rats diminished the aspirin-induced up-regulation of plasma PGE_2_ and IL-6, and significantly increased the expression of the anti-inflammatory IL-10 [86]. The levels of transforming growth factor β1 (TGF-β1), COX-2 and NO^●^ were also found lowered in the livers of fucoidan-fed alcoholic Balb/c mice compared to control animals [87]. 

More recently, a randomized controlled trial conducted with 122 patients suffering from osteoarthritis revealed that the daily intake of 300 mg of *F. vesiculosus* fucoidan for a 12-week period resulted in approximately 30% improvement of the patients’ knee joints. However, when compared to the placebo group, no significant differences were observed, which leads to inconclusive findings regarding to the possible fucoidan therapeutic effects for this chronic-inflammatory disease [88]. Nevertheless, a previous study, also carried out in osteoarthritis patients, revealed that the oral administration of Maritech, i.e., a commercial formulation composed of fucoidan-rich extracts from *F. vesiculosus* (85% *w*/*w*), *Macrocystis pyrifera* (10% *w*/*w*) and *Laminaria japonica* (5% *w*/*w*), over 12 weeks, significantly decreased the pain, stiffness, difficulty with physical activity and overall symptom severity in a dose-dependent manner [89]. Taking these data together, it is possible to suggest that a diet including fucoidan-rich foods may help prevent or attenuate inflammatory-related complications such as septicemia, intestinal bowel disease, alcoholic cirrhosis or osteoarthritis.

Promising anti-diabetic properties have also been reported for *Fucus* spp. fucoidans which have been described to exert acarbose-like properties due to their capacity to inhibit α-amylase and α-glucosidase activities, hence reducing the absorption of glucose into the bloodstream [90,91]. Additionally, fucoidan from *F. vesiculosus* was shown to increase the expression of GLUT-4, PPAR-γ and C/EBPα in 3T3-L1 adipocytes thus enhancing their sensitivity to insulin [92]. The anti-diabetic effects were also verified in vivo by different authors. In this context, Shan et al. [93] demonstrated that the oral administration of *F. vesiculosus* fucoidan decreased the fasting blood glucose and glycosylated hemoglobin levels of db/db mice [91]. In turn, feeding Goto–Kakizaki (GK) rats with fucoidan from this species caused the reduction of the post-prandial blood glucose while raising the serum levels of insulin. 

Anticoagulant and antitumor activities are two additional very well documented properties of fucoidans. Several in vitro studies have shown that fucoidans obtained from different species, including *F. vesiculosus*, *F. spiralis*, *F. serratus*, *F. distichus* and *F. evanescens*, displayed identical anticoagulant properties to that of heparin, i.e., a commercial animal-derived sulfated polysaccharide currently used as an anticoagulant drug, which are mainly mediated via activation of plasma antithrombin-III and inhibition of thrombin, heparin cofactor II and coagulation factor Xa [94,95,96,97]. Indeed, some in vivo studies have already shown promising results regarding the anticoagulant activity of *Fucus* spp. fucoidans [17,97]. However, none have been carried out using the oral administration as the delivery route, meaning that further investigation is necessary to understand weather dietary fucoidans can or not exert these effects in vivo.

As an antitumor agent, fucoidans may trigger one or multiple pathways depending on the target cancer cell lines. For example, in vitro antiproliferative effect of *F. vesiculosus* fucoidan towards melanoma B16 cells or human lymphoma HS-Sultan cells was shown to result from the triggering of caspase 3 [98,99], while on MCF-7 breast cancer cells, HeLa cervical epithelioid cells and HT-29 human colon cancer cells, apoptotic chain reaction was initiated by activation of caspases 8 and/or 9 [100,101,102]. In A549 lung carcinoma cells and Raji cells, *F. vesiculosus* fucoidan was found to suppress the expression of matrix metalloproteinase 2 and 9, respectively [103,104], while fucoidans from *F. serratus*, *F. distichus*, and *F. vesiculosus* were shown to block the formation of platelet-tumor aggregates, i.e., a mechanism of tumor cells transportation and fixation in the metastatic site, of a highly metastatic MDA-MB-231 breast cancer cells [96], thus preventing the tumor dissemination and metastization. Additionally, as *Fucus* spp. fucoidans were found capable of preventing human umbilical vascular endothelial cells (HUVEC) from entering in tubulogenesis (formation of capillary structures) [96] and even inhibit their vascular endothelium growth factor (VEGF)-induced proliferation, it is possible to suggest that these polysaccharides may also hold antiangiogenic potential [105].

Both anticoagulant and antitumor activities of fucoidans have been shown to be highly dependent of their structural features, particularly degree of polymerization (DP) or degree of sulfonation (DS) [106]. Indeed, it has been recently shown that the depolymerization of *F. vesiculosus* fucoidans progressively hampered their anticoagulant activity. Previous studies also showed that the anticoagulant properties of *F. vesiculosus* fucoidans was best for a DP above 200 units while procoagulant activity was observed for DP below 70 units [107]. Likewise, according to Cumashi et al. [96], the anticoagulant activities of *F. evansecens*, *F. serratus* and *F. distichus* were much lower compared with those of *F. vesiculosus* and *F. spiralis*, which had substantially lower DSs compared to the other three (29.2–36.3% *w*/*w* against 23.6–25.9% *w*/*w*, respectively). Supporting these results, further research demonstrated that desulfonation of *F. vesiculosus* fucoidans caused a negative impact on their anticoagulant activity [108]. On the other hand, the oversulfonation of fucoidan was shown to increase the anticoagulant activity in relation to its native form [77].

In turn, lower molecular weights seem to increase the antitumor effects of fucoidans. In this field, depolymerized *F. vesiculosus* fucoidans with 7–16 kDa revealed significantly higher cytotoxicity towards human stomach, human breast and human hepatocellular cancer cell lines and inhibition of TPA-induced neoplastic cell transformation than their native forms (with 217 kDa) [109]. Similar results have been reported for low molecular weight fucoidans from other seaweeds [110]. In fact, an in vivo study has already been carried out revealing that the combination of cyclophosphamide with medium molecular weight (20–40 kDa) fucoidan from *F. evanescens* significantly inhibits the number of metastases in C57BL/6 mice transplanted with Lewis lung carcinoma comparing to the administration of the drug alone [111]. In turn, native *F. evanescens* fucoidan but not their low molecular weight fragments exhibited anticancer activities in both human malignant melanoma cell lines SK-MEL-28 and SK-MEL-5, which was attributed to the lower sulfonation degree of the low molecular weight fragments compared to the native fucoidan [112]. Indeed, the treatment of B16 melanoma cells, Sarcoma-180 and Lewis lung carcinoma cells with oversulfonated fucoidans from *F. vesiculosus* revealed better anti-proliferative effect than the treatment with native fucoidans, thus emphasizing the importance of this structural feature for the antitumor activity of these polysaccharides [15].Imunomodulatory [113] anticomplementary [114] or anti-angiogenetic [115] activity are examples of other bioactivities described for fucoidans for which molecular weight and sulfonation degree have been shown to be determinant. 

Upon ingestion of fucoidan-rich foods, the cells that will be directly exposed to these polysaccharides are those of the gastrointestinal tract. Therefore, it is more likely that the antitumor activity of fucoidans will be further pronounced in these cells. Several authors reported effective antiproliferative activity of *F. vesiculosus* fucoidan against different human colon and stomach cell lines including HT-29, HCT-15 and HCT-116 human colon cancer cells, and MKN45 and Hs6 77.st human stomach cell lines [102,116,117]. Moreover, Vishchuk et al. [118] showed that the oral administration of different concentrations of *F. evanescens* fucoidan to xenograft mice models implanted with HCT-116 cells caused a significant dose-dependent inhibition of the tumor growth up to 72%. According to the authors, this chemopreventive effect was associated to the inhibition of the phosphorylation of ERK_1/2_ and histone H3, which are direct downstream signaling targets of lymphokine-activated killer T-cell-originated protein kinase (TOPK), that in turn is highly up-regulated in many cancers such as lymphoma, leukemia, melanoma, colorectal, breast, lung cancer, and cholangiocarcinoma. Additional in vivo studies carried out in different models of colon, hepatocellular, sarcoma and leukemia cancer cells have also described positive effects for oral ingestion of fucoidans, although these were not from *Fucus* spp. origin [119,120,121].

Anti-viral activity is another well-documented property of fucoidans. Indeed, as Ahmadi et al. [122] reviewed, fucoidans were shown to possess a remarkable capacity to inhibit a broad spectrum of viruses including poliovirus III, adenovirus III, ECHO6 virus, herpes simplex virus (HSV), citomegalovirus (CMV), dengue virus and even human immunodeficiency virus (HIV). However, many of those studies have been carried out using fucoidans from species non-related to the genus *Fucus*. Nevertheless, Queiroz et al. [123] showed pronounced in vitro anti-HIV activity for *F. vesiculosus* fucoidan which was closely related to its sulfonation degree. More recently, fucoidan extracted from *F. vesiculosus* has been tested against HSV-1 showing potent antiviral activity for the fucoidan fraction with the highest sulfate content [124]. Moreover, treatment of HBV-transfected hepatocytes with *F. vesiculosus* fucoidan showed a dose-dependent inhibitory effect on the virus DNA replicative intermediates through enhancement of ERK pathway and type I interferon response activation. These anti-HBV effects were further corroborated in vivo on HBV-infected mice models. However, the route used in this study for fucoidan administration was through intraperitoneal injection, thus further work is necessary to improve the understanding of the potential anti-HBV effect of these polysaccharides upon oral ingestion [125]. In this sense, to better comprehend the pharmacokinetic and tissue distribution of orally ingested fucoidan, Pozharitskaya et al. [126] has recently performed for the first time a study in which single-dose of 100 mg/kg body weight of *F. vesiculosus* fucoidan was supplied to male Wistar rats via intragastrical administration. According to their observations, the accumulation of fucoidan occurs preferentially in kidneys with a maximum concentration (C_max_) = 1.23 µg/g (after 5 h) and tissue availability (f_t_) = 10.85, followed by spleen (C_max_ = 0.78 µg/g after 3 h and f_t_ = 6.96) and liver (C_max_ = 0.53 µg/g after 2 h and f_t_ = 3.29), showing a relatively long absorption time and extended circulation in the blood, with a mean residence time (MRT) of 6.79 h.

Other less documented benefits described for *Fucus* spp. fucoidans include anti-obesity [92], anti-photoaging [127,128] and antibacterial [129,130].

#### 2.1.3. Laminarans

Laminarans are algal glucans that serve as reserve compounds. Although these are characteristic from Laminariales (up to 33% DW) [39], considerably high amounts of such polysaccharides can also be found in Fucales. In fact, *F. serratus* has been described as the non-*Laminaria* species with the highest content in these polysaccharides, which can amount up to 19% DW [131]. These glucans consist of small linear polysaccharides with an average molecular weight of 5 kDa built up of 20–50 glucose residues linked by β-(1→3)-glycosidic bonds, with β-(1→6)-branching points occurring randomly. Laminarans can be classified in two types depending on their polymeric chain, i.e., G-laminarans, containing only glucose residues, and M-laminarans containing a D-mannitol residue at the terminal reducing end (Figure 2F,G) [132]. In addition, according to these polymeric types and particularly the ramification degree, laminarans may occur in soluble or insoluble forms, the former totally soluble in cold water and the later only in hot water. Higher ramification degrees normally correspond to better solubility [133]. Variations in the structures of these polysaccharides are also observable from species to species, differing in terms of molecular weight, branching degree and M:G ratio [134].

Since these polysaccharides do not form viscous solutions nor gels, their applications in an industrial scale is still very limited [131]. Nevertheless, commercial interest of laminarans (or derivatives) is emerging due to their interesting bioactivities, such as antioxidant, antitumor, antimicrobial, immunomodulation and anticoagulant properties, that have recently been reported in literature, thus indicating that these polysaccharides might have promising potential to be used with medical/pharmaceutical purposes [39,135,136,137].

The number of works focusing the bioactivities of *Fucus* laminarans is, however, very limited until the moment. Still, a laminaran fraction extracted from *F. evanescens* was shown to possess mild antioxidant activity, exhibiting a total antioxidant capacity of 51 ascorbic acid equivalents/g DW [43]. More recently, Malyarenko et al. [44] reported that among three different laminarans isolated from *Saccharina cichorioides*, *Saccharina japonica* and *F. evanescens*, the later displayed the strongest antitumor activities against human colorectal adenocarcinoma, melanoma, and breast adenocarcinoma cells, showing 35–50% inhibition of cell proliferation, 35–60% inhibition of colony formation and 40–86% inhibition of cell migration at 200 μg/mL. Furthermore, the authors also noticed that higher inhibitory percentages could be achieved if sulfated derivatives of *F. evanescens* laminaran were used instead of the native form. Either forms revealed high antitumor activity against breast adenocarcinoma cells, followed by colorectal adenocarcinoma and melanoma, suggesting that *F. evanescens* laminaran and/or sulfated derivatives might be particular effective if used in breast cancer therapies [44]. Studies with laminarans extracted from other algal also pointed out the potential of these polysaccharides to act as antitumor agents [136,138]. Nevertheless, these are still very preliminary studies and further research is required in order to clarify whether dietary laminarans display such bioactivities.

### 2.2. Proteins and Amino Acids

The protein fraction of brown seaweeds is generally low and very susceptible to seasonality, with the highest contents being observed at the end of winter and during the spring period [139]. Among *Fucus* spp., *F. serratus* is the species that has shown the maximal protein content of 17% DW, while *F. vesiculosus* and *F. spiralis* accumulate up to 11% of protein (Table 1). These values are substantially low when compared to red and green algae (up to 44% DW) or even other genus of brown algae such as *Undaria*, for which protein content has been described to reach up to 24% DW [39]. Nevertheless, it is still high when compared to raw peas or fava beans (6.4% and 7.4% DW, respectively) which are commonly considered to be high vegetable protein suppliers [140].

The amino acids profile is also an important aspect to be considered since each individual amino acid may represent different roles in the organism. For example, aspartic acid and glycine are responsible for the formation of new tissues and regulations of the nervous system, while lysin and isoleucine are important for the immunologic system and phenylalanine for the thyroid function [141]. The most prevalent amino acids in *Fucus* spp. are undoubtedly aspartic and glutamic acids (Table 3) which according to Munda [142] may represent 22–44% of the total amino acids in the genus. The high content of glutamic acid is actually very important for the development of the special flavor and taste of seaweeds since this amino acid is the main responsible for the taste sensation of umami [143]. Alanine has also been seen in representative abundance in *F. evanescens* ethanolic extracts [144].

Moreover, seaweeds belonging to this genus contain all nine essential amino acids. The total content of these amino acids varies between 35% and 63% of total amino acids in *F. vesiculosus* and *F. spiralis*, respectively [24,142]. Threonine and valine, are abundant in *F. spiralis*, leucine in *F. vesiculosus* and *F. serratus*, and lysine in *F. serratus*. Together, these four amino acids represent the most abundant essential amino acids in *Fucus* spp., while the most limiting ones are methionine (except in *F. spiralis*), histidine and phenylalanine (Table 3). Few studies have documented the amino acid profile of different *Fucus* spp. which is resumed in Table 3. However, note that the amino acid content is very susceptible to seasonal variations, environmental conditions and other factors which are not contemplated in this table.

Recently, Paiva and co-workers [24] have reported an amino acid profile for *F. spiralis* that do not fit within the patterns above mentioned. In their work, glutamic acid was found in high abundance but not as the most abundant amino acid. Instead, leucine and isoleucine were the amino acids with higher prevalence, both accounting for about 20% of the total amino acids fraction. Other amino acids such as serine, proline arginine, threonine, valine and lysine were also found in relevant concentrations. 

### 2.3. Lipids and Fatty Acid Profile

Total lipid content in seaweeds is generally low, reaching up to 6% DW, which in a practical sense makes their contribution as food energy sources negligible [147]. The total lipid content in *Fucus* spp. ranges 0.4–5% DW, fitting in the aforementioned values (Table 1). *F. spiralis* was the species for which the highest lipid content was reported (5% DW) [24], while the lowest value was observed in *F. serratus* (0.4% DW) [28]. Note that, similarly to other biochemical components, the total lipid content of *Fucus* spp. may vary according to the season, environmental conditions, genetic differences and several other factors. In fact, the total lipid content in seaweeds has been reported to be higher during the winter and spring, and lower during the summer [148]. Nevertheless, *F. serratus* does not seem to fall within this pattern since its lipid content was reported to be higher in the summer [28]. Similar observations have been recently reported by Schmid et al. [149], who observed that the total lipid content in *F. vesiculosus* and *F. serratus* is higher in August rather than in November. 

Although they are low in fat, many authors have focused their studies in the lipid fraction of *Fucus* spp. due to the high content in polyunsaturated fatty acids (PUFAs) (Table 4), which are essential fatty acids of the utmost importance for human metabolism [150]. Since these fatty acids are not synthesized by mammals, they can only be introduced in our organism through diet [151].

From the reported studies, it is possible to conclude that the total content of PUFAs in *Fucus* spp. ranges between 24% and 48.5% of total fatty acids (TFA), with particular emphasis on *F. distichus* which is at the top of the list for about 10% more PUFA content than the remaining species. On the other hand, the total content of saturated fatty acids (SFAs) is maximum in *F. spiralis* reaching up to 53% TFA, while the amounts in other species range from 24% to 35% TFA. In contrast, *F. spiralis* is also the one with the lowest content in monounsaturated fatty acids (MUFAs) together with *F. distichus* (15.4–27.1% and 18.5% TFA, respectively).

The most abundant SFAs in the genus *Fucus* are palmitic (C16:0) followed by myristic (C14:0) acids, representing 9.6–19.6% and 1.3–15.5% of the TFA, respectively (Table 4). An exception to this is the palmitic acid content in *F. serratus*, which is substantially higher (up to 29% TFA) [28]. On the other hand, oleic acid (C18:1, ω9), not only is the major MUFA but also the major fatty acid of the genus reaching concentrations that may stand for almost 50% of the total fatty acids [155]. In fact, according to the review of Holdt and Kraan [39], *Fucus* spp. are among the marine macroalgae with the highest content on this fatty acid.

As mentioned before, PUFAs are of particular interest due to their favorable effects on human’s health, especially when considering ω3 fatty acids. Note that ω3 fatty acids have been recognized to exhibit anti-inflammatory and antioxidant activities, which may contribute to their beneficial cardiac effects while most ω6 fatty acids (precursors of arachidonic acid and prostaglandin E_2_) tend to promote inflammation [157]. It is therefore important to maintain an appropriate balance between ω6 and ω3 in the diet to reduce the risk of developing several diseases and maintain a healthy state. According to Simopoulos et al. [158], the optimal ratio of ω6/ω3 is 2–5:1, but, in the Western population, due to high consumption of refined oils rich in ω6, the actual ratio is 15–17:1.

Marine macroalgae are particularly abundant in ω3, which may represent up to 50% of their total fatty acids content [2]. In *Fucus* spp., the total content in ω3 ranges 6.4–25.7% which is not very different from the ranging concentrations of ω6 (9.6–24.9% TFA; Table 4). Therefore, the ratios between ω6 and ω3 found in this genus (0.7–3.7) are within or lower than the optimal limits aforementioned. The most prevalent ω3 fatty acids are eicosapentoic (C20:5, ω3) and stearidonic (C18:4 ω3) acids, ranging 1.1–15.8% and 0.8–11.1% of TFA, respectively [28]. Eicosatrienoic acid (C20:3, ω3) has also been reported in abundant concentrations (11.7–14.8% TFA) but only in *F. spiralis* [24,37]. In contrast, arachidonic (C20:4, ω6) and linoleic (C18:2, ω6) acids are the dominant ω6 fatty acids of this genus, reaching up to 16.4% and 14.2% TFA, respectively [28]. Relevant amounts of dihomo-γ-linolenic (14.3%; C20:3, ω6), an isomer of eicosatrienoic acid, were described also solely in *F. spiralis* [24]. 

In addition to fatty acids, the lipid fraction of *Fucus* spp. contains other interesting unsaponifiable compounds such as carotenoids, with special emphasis of fucoxanthin, which may range from 340 to 730 mg/kg DW [39]. Fucoxanthin has been described by several authors for its numerous interesting bioactivities with particular interest for application in nutraceutical and pharmaceutical fields. These properties will be discussed further in below.

### 2.4. Minerals

Algae content in minerals is generally high (8–40% DW), mostly due to the characteristics of their cell surface polysaccharides that allow them to retain inorganic marine substances [2]. Nevertheless, the mineral content of seaweeds is very variable according to the geographic harvesting site, wave exposure, seasonality and even between species in the same phylum [159]. According to Rupérez [160], the mineral content in brown algae is higher than in Rhodophyta. In the particular case of *Fucus* spp., the ash (mineral) content may vary from 19% to 36% DW (Table 1), which is substantially higher than the values found in terrestrial plants. Indeed, mean ash content of most common vegetables is much lower compared to that of *Fucus* spp. (e.g., 10.4% DW in potatoes, 7.1% DW in carrots and tomatoes, and 2.6% DW in sweet corn) [3]. 

Similar to other seaweeds, *Fucus* spp. contains several important minerals including Na, K, Ca, Mg, Fe, Cu, Zn, Mn and I [161]. Sodium and potassium are the major elements described in this genus, with concentrations ranging from 630 to 5469 and 976 to 4322 mg/100 g DW, respectively (Table 5). This represents up to eight times more sodium than cheddar cheese and 11 times more potassium than bananas [162]. However, despite these high levels, the Na/K ratio is actually low (0.6–1.5). Note that intakes of high Na/K ratios typical of modern Western diets are well known to be related with higher incidence of hypertension [163] and, therefore, products with low Na/K ratios may help to regulate the dietary Na/K imbalance. Indeed, this characteristic is one of the main advantages for the use of *Fucus* spp. and other seaweeds as foods. 

*Fucus* spp. also contain levels of Ca and Mg in much higher concentrations than those found in common foods. As an example, according to Ross et al. [166], the values of Ca in *F. serratus* may reach the 2175 mg/100 g DW, which represents almost 20 times more the Ca present in 100 g of whole milk [162]. Regarding Mg, the highest concentrations were described for *F. vesiculosus* corresponding to 994 mg/100 g DW [160], which represents approximately five times more the levels present in peanuts [162]. Particularly high levels of Fe have been described in *F. vesiculosus* and *F. spiralis* (up to 49 and 52 mg/100 g DW, respectively), while *F. serratus* seems to be a good accumulator of Mn (Table 4).

Exceptionally high amounts of iodine can accumulated in brown algae, and *Fucus* are no exception. In fact, *F. vesiculosus* along with *Laminaria sp.* were the original sources of iodine, found in 1811 by Courtois [13]. The content of this mineral in *Fucus* spp. may vary between 13 to 73 mg/100 g DW, with the maximal concentration being described in *F. vesiculosus* [168]. Indeed, according to Moro and Basile [169], this element is the most important active principle of this species since it has an active role in the production of thyroid hormones which in turn are responsible for the increase of the metabolism in most tissues, and the consequent raise of the basal metabolic rate [13]. Because of that, *F. vesiculosus* supplements are commonly used not only for the treatment of obesity but also for treating iodine deficiency goiter [170]. Indeed, the market is currently full of numerous *Fucus*-based supplements and nutraceuticals including InSea2™ (INNOVACTIV Inc., Rimouski, QC, Canada), Fucus Plus^®^ (North America Pharmacal Inc., Wilton, CT, USA), ThyroMate™ (Vitus Laboratories, LLC., College Park, MD, USA), SeaVeg^®^ (FarmaSea^®^, Scottsdale, AZ, USA) among many others, which are recommended for the treatment of these two complications. However, the consumption of such supplements must have some precautions since the daily intake of more than 600 μg of iodine (tolerable upper intake level for adults) may result in poisoning effects [171]. Nevertheless, considering that iodine deficiency is a reality at least in 11 European countries and most of the remaining countries are using iodized salts to control this problem, the introduction of *Fucus* spp. in population eating habits could be a valid alternative to ensure the intake of the optimal daily requirement of iodine and hence, a proper thyroid function [172].

The high bioaccumulative capacity of brown seaweeds may also be their worst disadvantage as the concentrations of heavy metals can reach toxic levels. This is particularly true for *Fucus* spp. since these belong to the Fucacea which is one of the most important biosorbent families from Phaeophyta [173]. Because of their inability to regulate the concentration of heavy metals in their tissues and also due to their tolerance to high external levels of such pollutants, seaweeds from this genus have been commonly used as bioindicators and bioremediators of heavy metal contaminated waters [174,175]. According to several authors, the content of As, Pb, Cd and Hg in *F. vesiculosus* growing in heavy metal contaminated waters may reach up to 73, 12, 10 and 2 mg/kg DW, respectively [176,177,178], representing 52, 2, 5 and 4 times more than the maximum permitted concentrations established for each element [179]. There is, however, a relation between the toxic effect of the heavy metals and their physical state, especially for As, whose inorganic forms are much more toxic than the organic ones [180]. Studies have shown that many algae, including *Fucus* spp., are able to metabolize inorganic arsenic into organic forms, and although this genus is naturally prone to accumulate substantial levels of arsenic, this is found predominantly in its organic form [181,182,183]. Therefore, even though *Fucus* spp. might have high levels of As, it does not necessarily mean that its consumption will cause poisoning effects. Nevertheless, before the consumption of these algae, it is always necessary to ensure that they meet the appropriate criteria concerning the heavy metal composition.

## 3. Other Nutritional Elements

### 3.1. Vitamins

Seaweeds are also a wealthy source of a broad range of these micronutrients which include water- (vitamin C and those belonging to the complex B) and fat-soluble (vitamin A, D, E and K) vitamins [184]. In fact, according to Chapman and Chapman estimations, the consumption of only 100 g of seaweed provides more than the daily requirement of vitamin A, B_2_ and B_12_ and two thirds of the vitamin C requirement [51].

**Figure 3:**
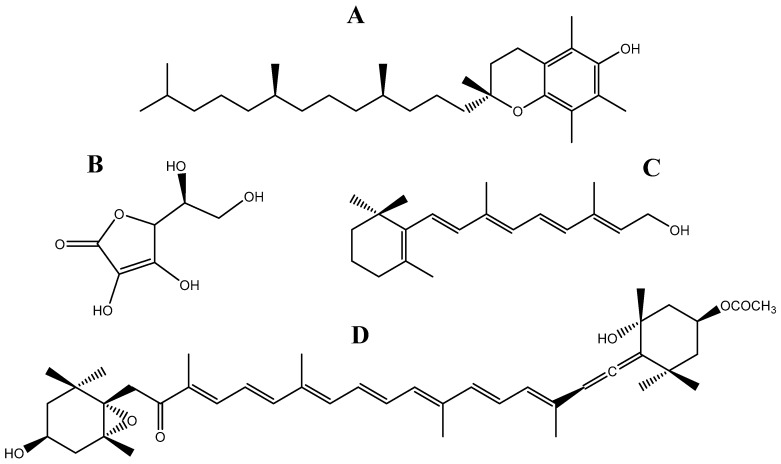
Representative chemical structure of: (**A**) vitamin E; (**B**) vitamin C; (**C**) vitamin A; and (**D**) fucoxanthin. Structures redrawn from [3,186,187,188]

Vitamins C and E (Figure 3A,B) are by far the most predominant in *Fucus* spp. The levels of vitamin C in *F. vesiculosus* are very variable, ranging from 141 to 770 mg/kg DW [185]. Such wide range of concentrations are a consequence of the variations in environmental conditions, geographical origin, light exposure, and, especially, seasonality. This has been demonstrated in different algal species including in *F. vesiculosus* and *F. serratus*, which exhibited minimum vitamin C concentrations during the winter (13 and 11 mg/100 g fresh weight (FW), respectively), while maximum concentrations were observed in the summer (77 and 48 mg/100 g FW, respectively) [51]. This means that a portion of *Fucus* spp. may contain identical or even higher amounts of this micronutrient than oranges (53 mg/ 100 g FW), which are the vitamin C-rich foods of excellence [184]. 

The positive effects that this vitamin has on human’s health have been extensively studied. It is believed that it may help to reduce the risk of cancers, regulate blood pressure and stimulate intestinal Fe absorption [189,190], but the main physiological role of vitamin C is as an antioxidant agent, participating in the ascorbate-glutathione cycle to reduce H_2_O_2_ to water as well as directly scavenging ^1^O_2_, O_2_^●−^ and OH^●^ [191]. Moreover, it also contributes for the recycling of tocopherol [192], which is another strong antioxidant vitamin involved in the prevention of lipid oxidation, protecting the membranes, fatty acids and lipoproteins from reacting with reactive oxygen species [193]. The levels of this vitamin in *Fucus* spp. may reach up to 600 mg/kg DW [194] making this genus one of the richest in vitamin E. *F. serratus* has been described as the species with the highest content in this micronutrient (600 mg/kg DW), followed by *F. spiralis* (511 mg/kg DW) and *F. vesiculosus* (490 mg/kg DW) [24,194], presenting approximately four times more vitamin E than olive oil (140 mg/kg), which is considered the major source of tocopherol in European’s diet [184]. Similar to other nutrients, vitamin E is strongly affected by seasonal fluctuations, however, unlike vitamin C, the periods of higher accumulation of this vitamin coincide with autumn and early winter, while the lowest concentrations are observable in spring [24,194]. From the different forms of vitamin E, α-, β-, δ and γ-tocopherols have all been described in *Fucus* spp., although α-tocopherol is usually the dominant form found in these seaweeds [24,195]. This is an important detail considering that α-tocopherol is the most active form of this vitamin [196]. Recently, it has been discovered that *F. vesiculosus* also contains trace amounts of three other vitamin E vitamers, namely α-, δ and γ-tocotrienols [197].

The levels of retinal, i.e., vitamin A (Figure 3C), an essential vitamin for the promotion of general growth, maintenance of visual function, regulation of differentiation of epithelial tissues, and embryonic development [198], are quite low in the genus *Fucus*, being described to reach up 14.1 mg/kg DW in *F. spiralis* and even less in *F. vesiculosus* (3.1 mg/kg DW) [14,24]. Nevertheless, this genus is endowed with considerable high amounts of β-carotene (provitamin A), which can be converted in retinal by the intestinal enzyme β-carotene 15,15′-monooxygenase [199]. The abundance of this carotenoid in *Fucus* seems to be maximum in *F. evanescens*, accounting for 32.1 mg/kg fresh weight, followed by *F. distichus* (21.3 mg/kg FW) and *F. spiralis* (6.9 mg/kg FW) [200]. In *F. vesiculosus* and *F. serratus*, the content of provitamin A may range 80–95 and 80–173 mg/kg DW, respectively [39]. Other vitamins including vitamin D, K, and some of the B-complex have also been described in *Fucus* spp., however, they have only been found in trace amounts [11,24].

### 3.2. Fucoxanthin

Fucoxanthin is the major carotenoid produced by brown algae and the main responsible for their brownish color [201]. This pigment belongs to the class of xanthophylls, possessing a unique structure, that includes an allenic bond, an epoxide and a conjugated carbonyl group in the polyene chain of the molecule (Figure 3D). Fucoxanthin may exist in *trans-* and *cis-*configurations, although the former is thermodynamically more stable than the latter [202].

According to Haugan et al. [203], approximately 70% of the total carotenoid content in *F. serratus* corresponds to fucoxanthin. Identical contents were further described either in *F. serratus* and *F. vesiculosus* [204]. Nevertheless, the content of this pigment in *Fucus* varies greatly from species to species and depending on the life cycle and environmental conditions. For instance, in *F. spiralis*, fucoxanthin accounts only for 171 mg/kg FW, while concentrations of 224 and 364 mg/kg FW were observed in *F. distichus* and *F. evanescens*, respectively [200]. Ramus et al. [205] reported concentrations up to 751 mg/kg FW in *F. vesiculosus* collected from shaded intertidal zones, which is much higher than the concentrations of other fucoxanthin-rich seaweeds including *A. nodosum* (172–660 mg/kg) or *Laminaria* spp. (178–468 mg/kg) [39]. However, these values dropped for 247 mg/kg FW in seaweeds growing in more sunny areas, representing a decrease of almost 70% in the fucoxanthin content. In addition to light exposure, these authors also observed that depth could be another determinant factor in the accumulation of this xanthophyll, since *F. vesiculosus* seaweeds growing at 4 m depth had 50% higher content in fucoxanthin than those growing in shallower areas. 

This xanthophyll has earned particular attention in the recent years mainly because of its promising anti-obesity effects, since it has the capacity to reduce the expression of adipocyte marker proteins such as PPARγ and C/EBPα, consequently inhibiting lipid uptake [206]. In addition, mRNA expressions of important lipid metabolism-related enzymes including hepatic acetyl-CoA carboxylase (ACC), fatty acid synthase (FAS), glucose-6-phosphate dehydrogenase (G6PDH), hydroxy-3-methylglutaryl coenzyme A (HMG-CoA), acyl-CoA cholesterol acyltransferase (ACAT), and SREBP-1C were found decreased in rats on a high fat diet supplemented with fucoxanthin [207]. Several other in vivo studies have shown that fucoxanthin may as well mediate plasma adipokine levels, down-regulating the lipogenic enzyme activities involved in fat production while up-regulating fatty acid β-oxidation activity and uncoupling protein gene expressions, thus stimulating metabolic thermogenesis and consequently contributing for the reduction of their body weight [208,209,210,211]. 

Fucoxanthin has also been reported to prevent oxidative stress-related lipid peroxidation and DNA damage through scavenging of intracellular ROS and enhancement of intracellular defenses by up-regulation of Nrf2/ARE signaling pathway and its down-stream phase II detoxification and anti-oxidant enzymes [212,213,214,215,216], to inhibit the expression of different pro-inflammatory mediators such as TNF-α, IL-1β and -6, NO^●^, iNOS, COX-2 and, more important, nuclear factor-κB (NF-κB) [217,218,219]. It may also exert antitumor activity either by stimulating apoptosis through activation of cytochrome *c*, cleavage of caspases 8, 9, 7 and 3 and inhibiting the expression of anti-apoptotic proteins such Bcl-Lx and Bcl-2 [220,221,222], or by activating cell cycle arrest through cytostatic activity and consequently blocking the tumor growth [223,224,225]. Other promising properties previously described for this pigment include anti-diabetes, anti-photoaging, anti-angiogenesis and anti-atherosclerosis effects [214]. 

Currently, fucoxanthin is considered safe by the European Food Safety Authority, Japan’s Food for Specified Health Uses, and the US Food and Drug Administration and has already been incorporated as functional ingredient in food products and supplements. In turn, medical and health applications have only emerged during the recent years. However, mostly due to its oxidative instability and the prohibitive costs of inefficient extraction methods, the utilization of fucoxanthin in industry remains underexploited [226]. Nevertheless, world fucoxanthin production in 2015 was approximately 500 t and it is expected to increase at least 5.3% per annum between 2016 and 2021 [227].

### 3.3. Phenolic Compounds (Phlorotannins)

**Figure 4:**
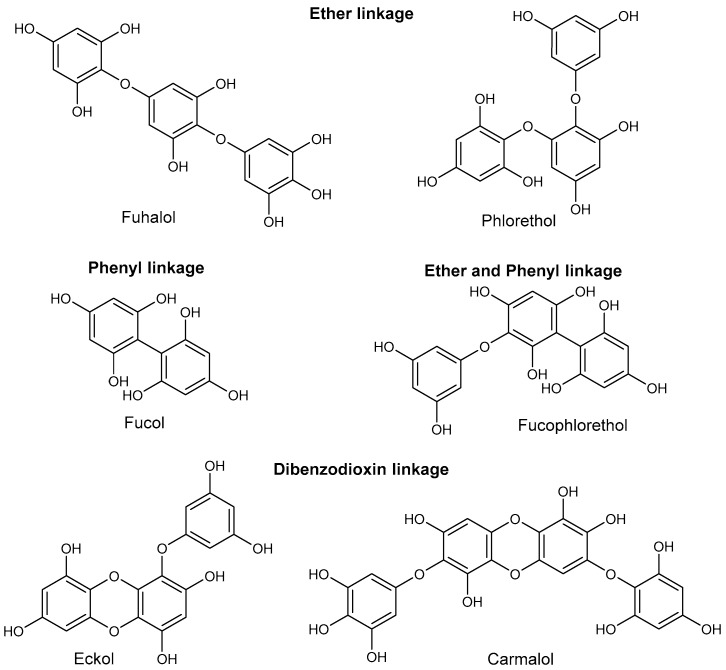
Examples of phlorotannin structures grouped according to their type of linkage. Structures redrawn from [251,252].

Phlorotanins are a group of marine exclusive phenolic compounds formed by polymerization of phloroglucinol (1,3,5-trihydroxybenzene) units, characteristically abundant in brown seaweeds. These are very hydrophilic compounds, biosynthesized through the acetate–malonate pathway, and their molecular weights vary between 126 Da and 650 kDa [228]. Depending on the nature of the structural linkages between phloroglucinol units and number of hydroxyl groups, phlorotannins can be grouped into fuhalols and phlorethols (possessing an ether linkage), fucols (possessing a phenyl linkage), fucophlorethols (possessing an ether and phenyl linkage), and eckols and carmalols (possessing a dibenzodioxin linkage) (Figure 4) [229]. From these, fucols and fucophlorethols are the predominant compounds found in *Fucus* spp. [230,231,232,233,234].

According to what Holdt and Kraan reviewed, the highest phlorotannin content recorded for a *Fucus* sp. was 12% DW [39]. However, the accumulation of phlorotannins in this genus may vary from species to species, as it was observed by Connan et al. [235], who reported that *F. vesiculosus* had the highest phlorotannin content (approximately 6% dry weight) compared to *F. serratus* and *F. spiralis* (4.3% and 3.9%, respectively). Furthermore, other factors such as seasonality, solar exposure or salinity may contribute as well for significant intra-species variations. Indeed, the phlorotannin concentrations of *Fucus* spp. have been reported to be higher during the summer [235] at higher salinity waters [236,237] and solar exposure periods [238].

During the last few years, these compounds have gathered great interest since they have been shown to exert numerous biological activities with potential application in food, pharmaceutical and cosmetic industries, and others. As phenolic compounds, the antioxidant activity is the greatest potential of phlorotannins, with no exception for those from *Fucus* origin. Indeed, these have been described as having great capacity to scavenge different radicals [239,240,241,242], prevent oxidative stress-induced DNA damage and stimulate cellular antioxidant defenses [213,243]. Interestingly, although Wang et al. [242] previously reported that there was no evident correlation between antioxidant activity and the molecular weight of phlorotannins extracted from *F. vesiculosus*, it has been recently demonstrated that these are indeed correlated since the antioxidant activity of phlorotannins from this species tend to decrease with the increase of their degree of polymerization, thus indicating an inversely proportional correlation [244]. 

Phlorotannis extracted from *Fucus* spp. have also been described for their good anti-inflammatory effects by counteracting an array of pro-inflammatory mediators which include several cytokines such as IL-1β, IL-6, IL-17 and TNF-α, as well as chemokines such as monocyte chemoattractant protein-1 (MCP-1), macrophage inflammatory protein 2α (MIP-2α), interferon gamma-induced protein 9 and 10 (IP-9 and -10), enzymes iNOS and COX-2, toll-like receptors 4 and 9, components of NF-κB, adhesion molecules and many others [213,245,246,247]; and good antidiabetic effects, exhibiting acarbose-like activity, i.e., acting as α-amylase and α-glucosidase inhibitors and consequently reducing the polysaccharides breakdown, glucose absorption and postprandial hyperglycemia [248,249]. In fact, the antidiabetic activity of *F. vesiculosus* was even demonstrated in a human trial in which a single ingestion of 250 mg of InSea^2^™ (a commercial extract of *F. vesiculosus* and *Ascophyllum nodosum*, INNOVACTIV Inc., Rimouski, QC, Canada), 30 min prior to the consumption of 50 g of carbohydrates, caused a reduction of the insulin incremental area of the curve by 12.1% and an increase of the insulin sensitivity by 7.9% [250].

Other potential biological properties of *Fucus* spp. phlorotannins include antitumor [253,254], antimicrobial [255,256], anti-hypertensive [257], anti-obesity [245] and photoprotective [258], although studies focusing these bioactivities are still incipient.

## 4. *Fucus* spp. as a Functional Ingredient

Although the term “functional foods” keeps earning more and more popularity worldwide, there is still no clear definition for it. Nevertheless, these are commonly accepted as foods (and food components) that provide specific health benefits beyond the basic nourishment. Generally, these foods are intended to be consumed as part of the normal diet and contain biologically active components which offer the potential of enhanced health or reduced risk of disease [259].

Since seaweeds have proven to possess promising health beneficial effects, they appear to be good candidates for application as functional ingredients in the design of novel functional foods. In fact, according to the SeafoodSource report, the new products containing this “new ingredient” launched on the European market between 2011 and 2015 increased by 147%, making Europe the most innovative region globally after Asia Pacific [260]. In this field, the use of *Fucus* spp. and/or its extracts for development of food with improved nutritional qualities and functionalities have already taken some steps (Table 6).

In this context, O’Sullivan et al. [22] used *F. vesiculosus* ethanol 60% extract at 0.25 and 0.5% (*w*/*w*) to produce a novel dairy product. The authors observed that when incorporated at the highest concentration, the extract provided antioxidant functionality in milk either before or after in vitro digestion. The ability was however not observed in cellular models, suggesting that fortification with *F. vesiculosus* could improve certain milk qualities and shelf-life characteristics, but did not provide significant biological activity. Furthermore, milk enriched with *F. vesiculosus* presented a green/yellowish color and fishy taste that was not generally well accepted in the sensorial evaluation. Further studies conducted by this research group on fortified yogurts with the same *F. vesiculosus* ethanolic extract (0.5% *w*/*w*) also revealed an increment of the antioxidant activity before and after in vitro digestion, without affecting product’s acidity, microbiology or whey separation parameters. However, once again, the biological activity on cellular models was absent and sensorial analysis had low acceptability [21]. 

Several authors also reported the use of *F. vesiculosus* extracts for development of novel fish and fish-derived food products. Indeed, the application of *F. vesiculosus* ethyl acetate extract to hemoglobin-fortified washed cod mince was found to effectively reduce the lipid and protein oxidation events in the food, suggesting that this seaweed extract could have great potential to be used as a natural antioxidant agent in fish muscle foods [262]. Similar studies further confirmed that the ethyl acetate fraction of *F. vesiculosus* extract not only could effectively prevent the hemoglobin-mediated lipid oxidation occurring during cod protein enzymatic hydrolysis as also significantly contributed for an enhancement of the fish protein hydrolysates antioxidant activity [265]. Additionally, this *F. vesiculosus* fraction was also shown to decrease the rancid, bitter, soap and fish oil tastes of cod bone mince-fish protein hydrolysates, thus contributing for their overall better tasting [264]. Nevertheless, due to the safety implications associated to the use of organic solvents in products for direct human consumption, the use of ethyl acetate extracts would be very limitative if considering their application as food ingredients.

In a recent study, the addition of *F. vesiculosus* aqueous or ethanol extracts to ω3 enriched fish cakes revealed that, although no significant differences were seen regarding to the cakes’ lipid oxidation or quality, no off-flavors and even lower rancid odor and flavor was noticed when comparing to the control cakes [261]. On the other hand, *F. vesiculosus* ethanol extracts and subsequent phlorotannin-purified fractions have shown promising results in two cod model systems, namely hemoglobin-mediated lipid oxidation in washed cod muscle and cod protein isolates. In fact, the use of different oligomeric phlorotannin subfractions (300 mg/kg) were even capable of completely inhibiting initiation of lipid peroxidation in both cod model systems throughout the entire study period (eight days), thus exhibiting an effect comparable to that of 100 mg/kg of propyl gallate, i.e., a highly effective synthetic antioxidant in muscle foods [263]. Diaz-Rubio et al. [20] also demonstrated that the addition of 1% or 2% of *F. vesiculosus* antioxidant dietary fiber to minced horse mackerel contributes to the reduction of fish’s lipid oxidation during its storage. However, even though the palatability of the 2% *F. vesiculosus* fiber-supplemented fish samples was not impaired, an alteration of the fish flavor was noticed in the sensorial analysis. 

As an alternative to the direct application of *Fucus* extracts into the fish matrix, studies have been carried out to evaluate the possible preservative effects of *Fucus* spp. when added to the icing medium conventionally used for storage and preservation of fresh fish. In this field, the work of Rodrigues [269] revealed that, although the ice supplementation with *F. spiralis* ethanolic extracts presented high antioxidant activity and stability over 60 days, its capacity to delay or inhibit the lipid oxidation of sardine fillets was very low. On the other hand, the use of a *F. spiralis*-supplemented ice medium for storage of megrim over 14 days significantly inhibited the microbial activity of several microorganisms including aerobes, psychrotrophs, proteolytic bacteria and lipolytic bacteria, as well as the pH raise and lipid oxidation of the fish samples compared to the control [270]. The main difference between these two studies is that, the sardine fillets were placed on top of the supplemented ice, while the megrim fishes were covered with the supplemented ice. Therefore, upon ice thawing, *F. spiralis* extract was washed over megrim fishes but not on sardine fillets, which could explain these contradictory results.

*F. vesiculosus* extracts have also been found to be particularly useful for preventing lipid oxidation in fish-oil-enriched foods. For instance, Karadag et al. [266] reported that the introduction of *F. vesiculosus* ethanol and acetone extract at 0.5 g/100 g exhibited the most efficient antioxidant effect and highest improvement of the bars lipid stability, while water extracts only exerted positive effects for the concentration 1.0 g/100 g. Previous studies revealed that the addition of 1.0–2.0 g/kg of a phenolic and carotenoid-rich fraction from a *F. vesiculosus* 80% ethanol extract to fish-oil-enriched milk dose-dependently enhanced its lipid stability as well [19]. On the other hand, despite its lower content of phenolics and pigments, the addition of 2.0 g/kg of a *F. vesiculosus* water extract to fish-oil-enriched mayonnaise exhibited the highest inhibitory effect against peroxide formation, possibly due to its higher metal chelating capacity and presence of other highly polar compounds than phenolic compounds [19]. In a similar study, the addition of 1.5–2.0 g/kg of ethanol or acetone *F. vesiculosus* extracts to fish-oil-enriched mayonnaise was also shown to cause a dose-dependent reduction of lipid oxidation reactions and hydroperoxides formation, however, the addition of water extracts exerted an opposite effect, i.e., acted as a pro-oxidant. This result could be a consequence not only of the lower phenolic and carotenoid content compared to the ethanol and acetone extracts but also due to the much higher content in trace metals, namely Fe, which could be stimulating the oxidative reactions [267]. Recently, aiming the substitution of the synthetic antioxidant 2,6-d-*tert*-butyl-4-methylphenol (BHT) conventionally used as a stabilizer in food products, Agregán et al. [268] developed a *F. vesiculosus*-fortified canola oil. According to the authors, after 16 days of accelerated storage conditions (at 60 °C), the canola oil fortified with 500 ppm of *F. vesiculosus* extract exhibited a reduction of the peroxides formation of approximately 70% compared to the control, which was much lower than values observed for canola oil fortified with 50 ppm of BHT (approximately 30%). Therefore, according to these studies, it is possible to propose that *Fucus* spp. extracts with high phenolic and/or carotenoid contents could be used not only as natural antioxidants for food preservation but also to replace the use of synthetic compounds commonly used for those purposes.

## 5. Concluding Remarks

*Fucus* spp. are very valuable food sources with high nutritional value and low in calories due to their low content of lipids and poor bioavailable polysaccharides. Although the nutrient content of this genus is highly dependent of seasonal, environmental, genetic and other variations, it is generally very rich in dietary fiber, constituting an important source of fucoidan and alginic acid. Furthermore, the mineral content of *Fucus* spp. is also very rich, surpassing that of common terrestrial vegetables. Algae from this genus generally contain high amounts of Na and K, although their ratio is low, which could be useful for the regulation of the Na/K imbalance in Western countries. Moreover, these algae can accumulate high concentrations of iodine which is deficient in almost half of the European population and is very important for the thyroid function. Although these macroalgae are not the abundant in protein when compared to red or green seaweeds, their protein levels are still higher than those of some terrestrial plants commonly considered as high vegetable protein suppliers, containing all nine essential amino acids and being particularly rich in glutamic and aspartic acids. Likewise, despite the low lipid content, *Fucus* spp. lipid profile is highly abundant in PUFAs displaying very low ω6/ω3 ratios contrary to what is currently observed in Western diets.

*Fucus* spp. algae are also a valuable source of bioactive compounds that could be explored for the development of natural agents with therapeutic applications. Among them, one can highlight the fucoidans and the phlorotannins, although there are others equally important such as vitamins and fucoxanthin. These compounds have drawn much attention during the recent years due to their numerous possible therapeutic properties. Common features among them are the good antioxidant effects through scavenging of ROS or enhancement of intracellular antioxidant defenses, good anti-carcinogenic effects through activation of apoptosis on cancerous cells and inhibition of metastasis and angiogenesis, and good anti-inflammatory effects through inhibition of several pro-inflammatory mediators. Other important biological activities have been demonstrated such as anti-obesity, anti-diabetes, anti-microbial or anti-photoaging activities, and, in the case of fucoidan, anti-coagulant/anti-thrombotic activities as well. Therefore, it can be suggested that not only *Fucus* spp. nutrition value could valuably contribute for the improvement of the dietary quality of Western countries but also the presence of compounds with such powerful and versatile bioactivities grant them great potential to be exploited as renewable feedstocks for extraction of natural active ingredients for development of new added-value products. Particularly in food industry, *Fucus* spp. has already demonstrated to be particularly useful for improvement of foods quality and shelf-lives, as well as for the development of novel functional foods. Nevertheless, numerous other applications could be given to this genus, namely in the field of nutraceutical, cosmeceutical and pharmaceutical products development. With this manuscript, we hope to raise the awareness for the multiple applications of *Fucus*-derived products, as well as to contribute to boost their commercial interest.

## Figures and Tables

**Table 1:** Moisture and macronutrients composition of different *Fucus* spp.

Species	Moisture	Carbohydrates	Fiber	Protein	Lipid	Ash	Ref.
*F. serratus*	80–81	26 *–62	16	10–17	0.4–3	19–22	[25,26,27,28,29]
*F. vesiculosus*	71–84	34–66	4–59	1–11	1.2–4	23–36	[18,27,29,30,31,32,33,34,35,36]
*F. spiralis*	82–88	63 *	63	10–11	1.8–5	22	[12,24,37]
*F. distichus*	N.D.	N.D.	N.D.	N.D.	3	N.D.	[38]
*Fucus* spp.	68–88	26–66	4–63	1–17	0.4–5	19–36	[2,20,39]

Values are expressed in % DW. * calculated by a differential method subtracting (protein + lipid + ash) from total dry weight; N.D., no data found in the literature.

**Table 2:** Polysaccharide composition of *Fucus* species (% DW). Adapted from [36,42,43,44,45,46,47,48,49,50,51,52,53,54,55,56,57,58].

Species	Fucoidan	Alginic Acid	Laminaran
*F. vesiculosus*	3.4–25.7	8.4–58.8	0.6–7.0
*F. serratus*	13.0–24.4	10.5–22.2	1.0–19.0
*F. spiralis*	N.D.	13.0–16.6	1.5–6.9
*F. distichus*	14.6–21.5	9.6–23.6	2.2
*F. evanescens*	0.8–18.0	0.3–20.0	0.4–2.7
*Fucus* spp.	0.8–25.7	0.3–58.8	0.4–19.0

Values expressed in % DW; N.D., no data found in the literature.

**Table 3:** Amino acid composition of different *Fucus* species. Adapted from [142,144,145,146].

Aminoacid ^a^	*F. vesiculosus*	*F. ceranoides*	*F. spiralis*	*F. serratus*	*F. evanescens* ^b^	*Fucus* spp. ^c^
EAA						
Thr	3.91–4.72	3.82	5.97	2.38	2.20	2.38–5.97
Val	3.98–4.49	3.54	6.50	5.96	1.80	3.54–6.50
Met	1.39–1.74	0.74	4.78	0.17	N.D.	0.17–4.78
Ile	3.06–3.91	2.77	4.49	4.48	N.D.	2.77–4.49
Leu	5.60–6.64	4.80	4.19	7.78	1.80	4.19–7.78
Phe	3.25–4.17	2.99	2.78	3.86	3.50	2.78–3.90
Lys	3.95–6.16	3.54	4.96	5.85	1.50	4.08–5.85
His	1.36–1.70	1.24	1.89	1.48	N.D.	1.24–1.89
NEAA						
Asp	7.98–12.91	9.88	12.05	13.85	4.50	7.98–13.85
Ser	3.76–4.85	3.58	6.79	5.96	1.20	3.58–6.79
Glu	9.10–24.28	31.47	16.30	24.46	26.00	9.10–31.47
Pro	3.33–4.43	2.76	3.01		5.00	2.76–3.71
Gly	4.06–5.01	3.63	10.16	6.75	2.20	3.63–10.16
Ala	4.93–7.59	6.08	11.05	11.58	32.00	4.93–11.58
Tyr	1.50–2.52	2.99	1.36	1.42	2.50	1.36–2.14
Arg	4.38–4.56	3.94	1.59	2.95	2.00	1.59–4.56

Values are expressed as g/100 g protein. ^a^ Trp and Cys are missing from this table because methods used by the authors do not allow to analyze the contents of these two amino acids. ^b^ Values for *F. evanescens* are expressed in % DW. ^c^ Range values in *Fucus* spp. column do not contemplate the values of *F. evanescens*. EAA, essential amino acids; NEAA, non-essential amino acids, N.D., no data found in the literature.

**Table 4:** Fatty acid profile of different *Fucus* species. Adapted from [24,28,37,38,149,152,153,154,155,156].

Fatty Acid	*F. vesiculosus*	*F. spiralis*	*F. serratus*	*F. virsoides*	*F. distichus*	*F. evanescens*	*Fucus* spp.
SFA	24.3–34.0	33.6–53.2	26.9–33.5	30.3–35.4	29.4	27.6	24.3–53.2
C10:0	2.8–18.8	3.2–12.9	N.D.	N.D.	N.D.	N.D.	2.8–18.8
C12:0	0.5–0.7	tr–1.0	0.3–0.7	N.D.	N.D.	N.D.	tr–1.0
C13:0	7.5–14.4	11.7	N.D.	N.D.	N.D.	N.D.	11.7
C14:0	12.4	1.3–15.5	6.4–10.9	14.2–14.9	8.4	11	1.3–15.5
C15:0	N.D.	0.3	N.D.	N.D.	0.6	N.D.	0.3–0.6
C16:0	9.6–17.7	13.6–18.8	18.9–29.2	12.3–14.9	19.6	15.8	9.6–29.2
C18:0	0.6–2.2	0.6–0.8	0.9–1.7	1.6–2.8	0.8	0.6	0.6–2.8
C20:0	tr	0.5	tr–0.5	2.1–2.9	N.D.	0.2	tr–2.9
C21:0	N.D.	0.6–7.6	N.D.	N.D.	N.D.	N.D.	0.6–7.6
C22:0	tr	tr–0.7	N.D.	N.D.	N.D.	N.D.	tr–0.7
MUFA	23.8–47.1	15.4–27.1	27.2–41.3	34.5–35.2	18.5	39.3	15.4–41.3
C14:1, ω5	0.2–1.3	0.1–0.6	0.3–0.9	N.D.	N.D.	N.D.	0.1–1.3
C16:1, ω7	0.9–1.5	1.1	tr–9.5	1.3–1.6	1.8	1.2	tr–9.5
C18:1, ω9	21.3–46.9	14.3–33.3	11.4–41.3	32.1–33.9	16.7	38.1	11.4–46.9
PUFA	25.8–42.5	31.4–39.0	28.2–39.4	24.2–29.7	48.5	29.9	24.2–48.5
C18:2, ω6	7.5–10.0	6.4–11.7	7.6–14.2	3.1–3.8	7.7	13.2	3.1–14.2
C18:3, ω6	2.7–5.0	tr–9.4	0.6–9.7	0.2–0.31.5–2.1	0.4	0.6	0.2–9.4
C18:3, ω3	N.D.				7.5	2.9	tr–7.5
C18:4, ω3	2.2–3.2	N.D.	1.5–11.1	0.8–1.3	6.7	1.2	0.8–11.1
C20:2, ω6	tr–0.9	0.3–0.4	tr	N.D.	0.6	N.D.	tr–0.9
C20:3, ω6	tr–1.0	14.3	tr	0.8–1.9	0.6	0.7	tr–14.3
C20:3, ω3	N.D.	11.7–14.8	N.D.	N.D.	N.D.	0.2	11.7–14.8
C20:4, ω6	7.4–13.1	0.4	9.1–16.4	9.6–15.1	14.1	8.7	0.4–16.4
C20:5, ω3	3.7–7.3	1.1–6.8	3.9–15.8	5.7–8.1	10.9	2.1	1.1–15.8
C22:6, ω3	0.7–2.1	1.3–3.3	0.8–3.6	N.D.	N.D.	N.D.	0.7–3.6
ω3	8.6–24.0	14.0–14.8	9–22.4	9.2–9.3	25.7	6.4	6.4–25.7
ω6	17.2–20.6	9.6–24.9	16.8–22.0	15–20.4	23.4	23.5	9.6–24.9
ω6/ω3	0.9–2.0	0.7–1.8	0.9–2.4	1.6–2.1	0.9	3.7	0.7–3.7

Values expressed in percent of total fatty acid. SFA, saturated fatty acids; MUFA, monounsaturated fatty acids; PUFA, polyunsaturated fatty acids; tr, trace; N.D., no data found in the literature.

**Table 5:** Major mineral and trace elements in different *Fucus* spp. Adapted from [24,154,160,164,165,166,167,168].

Element	*F. vesiculosus*	*F. spiralis*	*F. serratus*	*F. guiryi*	*Fucus* spp.
Na	630–5469	1429	2305	N.D.	630–5469
K	1100–4322	976	2490	N.D.	976–4322
Ca	938–2150	118–1049	1284–2175	895	118–2175
Mg	740–994	163–819	724–844	702	163–994
Fe	4.2–49	52	31	13	4.2–52
Cu	0.2–1.4	0.2	0.3–1.4	0.2	0.2–1.4
Zn	2.6–28	15	5.3–29	4.5	2.6–29
Mn	3.4–6.6	6.3	14–29	11	3.4–29
I	13–73	23.3	32	27	13–73
Na/K	0.6–1.3	1.5	0.9	N.D.	0.6–1.5

Data expressed in mg/100 g DW. N.D., no data found in the literature.

**Table 6:** Effect of functional foods enriched with *Fucus* components.

Functional Food	Functional Ingredient	Quantities	Results	Ref.
Milk	*F. vesiculosus* 60% EtOH extract	0.25% and 0.5%	- Extract was stable in milk and provided antioxidant activity before and after in vitro digestion, but not in cells - Improvement of certain milk quality and shelf-life characteristics - Milk sensorial attributes were worsened	[22]
Yogurt	*F. vesiculosus* 40% EtOH extract	0.25% and 0.5%	- No influence on product’s acidity, microbiology or whey separation parameters - Increment of the antioxidant activity before and after in vitro digestion - Improvement of certain yogurt quality and shelf-life characteristics - Yogurt sensorial attributes were worsened	[21]
Fish cakes	*F. vesiculosus* extracts: 100% H_2_O, 80% EtOH	3.7 and 3.8 g/100 g (H_2_O and EtOH extract, respectively)	- No off-flavors and lower rancid odor and flavor -None of the extracts had influence on lipid oxidation nor quality of the products	[261]
Cod muscle	EtOAc fraction of *F. vesiculosus* 80% EtOH extract	300 mg PGE/kg	- Application of seaweed extract acted against lipid oxidation in fish muscle foods.	[262]
Cod mince	*F. vesiculosus* 80% EtOH extract and further fractions (EtOAc + Sephadex LH-20)	300 mg/kg	- Phlorotannin-rich fractions had higher inhibitory impact on lipid peroxidation than crude 80% ethanol extracts - Some phlorotannin-rich sub-fractions had an effect comparable to that of 100 mg/kg propyl gallate.	[263]
Cod protein hydrolysates	*F. vesiculosus*: EtOAc fraction from an 80% EtOH extract	62.0 g PGE/100 g	- Decreased lipid hydroperoxide and TBARS values during protein hydrolyzation - Increased antioxidant activity of the final protein hydrolysates	[264]
Cod bone mince protein hydrolysates	*F. vesiculosus*: EtOAc fraction from an 80% EtOH extract	0.16 g PGE/L of 3.7% (*w*/*v*) cod bone mince	- Prevented lipid oxidation during protein hydrolysates freeze drying - Slightly increased antioxidant activity of the final protein hydrolysates - Improved the bitter, soap, fish oil and rancidity taste of the final protein hydrolysates	[265]
Minced horse mackerel	*F. vesiculosus* antioxidant dietary fiber	1% and 2%	- Prevented lipid oxidation during 5 months of storage at −20 °C. - Reduced total yield after thawing and cooking after up to 3 months of frozen storage	[20]
Fish-oil-enriched granola bars	*F. vesiculosus* extracts: 100% H_2_O, 70% ACN, 80% EtOH	0.5 and 1.0 g/100 g	- The highest antioxidant effect and lipid stability improvement was observed for EtOH and ACN extracts at 0.5 g/100 g. - H_2_O extract only showed positive effects for the concentration 1.0 g/100 g	[266]
Fish-oil-enriched milk and mayonnaise	*F. vesiculosus*: EtOAc fraction from an 80% EtOH extract, 100% H_2_O	1.0–2.0 g/100 g	- EtOAc fraction caused significant improvement of milk’s lipid stability in a dose-dependent fashion, decreasing the degradation of EPA and DHA and subsequent secondary degradation products - H_2_O extract at 2.0 g/100 g exerted higher inhibitory effects on mayonnaise’s peroxide formation. Lower concentrations had pro-oxidant effect	[19]
Fish-oil-enriched mayonnaise	*F. vesiculosus* extracts: 100% H_2_O, 70% ACN, 80% EtOH	1.5 and 2.0 g/kg	- The higher the concentration, the better the inhibitory effect of EtOH and ACN extracts against lipid oxidation. - Water extracts caused pro-oxidant effects, probably due to high contents of trace metals.	[267]
Canola oil	*F. vesiculosus* H_2_O extracts	500 ppm	- Peroxide, *p*-anisidine, total oxidation and conjugated dienes values decreased approximately 70% comparing to the control and 40% comparing to BHT - TBARS values decreased approximately 50% comparing to the control and 30% comparing to BHT	[268]

ACN, acetone; EtOH, etanol; EtOAc, ethyl acetate; PGE, phloroglucinol equivalents; BHT, butylated hydroxytoluene; CAN, acetonitrile.
